# Recovery of LiCoO_2_ and graphite from spent lithium-ion batteries by molten-salt electrolysis

**DOI:** 10.1016/j.isci.2023.108097

**Published:** 2023-09-30

**Authors:** Jin Feng, Beilei Zhang, Pin Du, Yahong Yuan, Mengting Li, Xiang Chen, Yanyang Guo, Hongwei Xie, Huayi Yin

**Affiliations:** 1 Key Laboratory for Ecological Metallurgy of Multimetallic Mineral of Ministry of Education, School of Metallurgy, Northeastern University, 11 Wenhua Road, Heping District, Shenyang 110819, P.R. China; 2 Key Laboratory of Data Analytics and Optimization for Smart Industry, Ministry of Education, Northeastern University, Shenyang 110819, P.R. China; 3 School of Materials Science and Engineering, Henan Normal University, Xinxiang 453007, P.R. China; 4 School of Resource and Environmental Science, Wuhan University, 299 Bayi Road, Wuhan, Wuchang District 430072, P.R. China

**Keywords:** Electrochemical energy storage, Engineering, Energy resources

## Abstract

The recovery of spent lithium-ion batteries has not only economic value but also ecological benefits. In this paper, molten-salt electrolysis was employed to recover spent LiCoO_2_ batteries, in which NaCl-Na_2_CO_3_ melts were used as the electrolyte, the graphite rod and the mixtures of the spent LiCoO_2_ cathode and anode were used as the anode and cathode, respectively. During the electrolysis, the LiCoO_2_ was electrochemically reduced to Co, and Li^+^ and O^2−^ entered into the molten salt. The O^2−^ was discharged at the anode to generate CO_2_ and formed Li_2_CO_3_. After electrolysis, the cathodic products were separated by magnetic separation to obtain Co and graphite, and Li_2_CO_3_ was recovered by water leaching. The recovery efficiencies of Li, Co, and graphite reached 99.3%, 98.1%, and 83.6%, respectively. Overall, this paper provides a simple and efficient electrochemical method for the simultaneous recovery of the cathode and the anode of spent LiCoO_2_ batteries.

## Highlights

• A molten-salt electrolysis method is developed to recover spent LiCoO_2_ batteries
• Both spent cathode and anode are recovered simultaneously
• The recovery efficiencies of Li, Co, and graphite reach 99.3%, 98.1%, and 83.6%

## Graphical abstract

**Figure:**
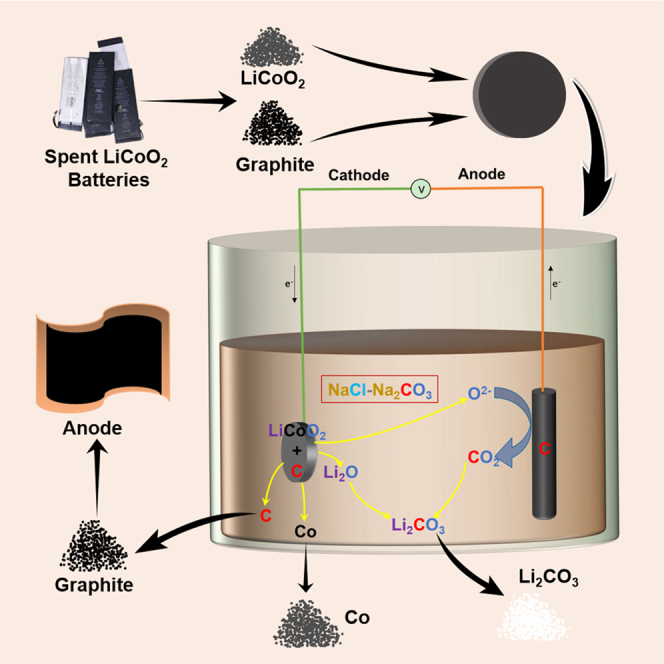

## Introduction

Lithium-ion batteries (LIBs) are clean energy storage devices, which are widely used in electronic products such as cell phones, notebook computers, digital cameras, and electric cars because of their advantages of high energy density, low memory effect, safety, and long life.^1^^,^^2^^,^^3^^,^^4^^,^^5^ Not only the widespread use of LIBs consumes a large number of metal resources (such as cobalt (Co) and lithium (Li)), but also spent LIBs pose a serious threat to human health, the atmosphere, water resources, and soil.^5^^,^^6^^,^^7^ The global battery markets are expected to exceed $100 billion by 2025.^8^ With the rapid growth of power battery production, the demand for the Co and Li will also continue to rise, and the supply and demand will be more tense. If properly recycled, spent LIBs will have huge economic value and become a veritable “urban mine”.

The LIBs’ recovery methods mainly include pyrometallurgy, hydrometallurgy, electrometallurgy, and biometallurgy. Pyrometallurgy recovery technology includes the reduction and melting of metal oxides, separation of metals with different boiling points, and combustion of organic chemicals.^6^^,^^9^^,^^10^^,^^11^^,^^12^^,^^13^ This method has the advantages of high chemical reaction rates, large processing capacities, relatively flexible raw materials, and simple operation. However, it would cause environmental pollution and consume a huge amount of energy.^14^ Hydrometallurgy includes pretreatment, leaching, separation, and recovery of precious metals from the leachate. The purpose of leaching is to convert the metals of the cathode material into ions in the solution. The leaching process is usually carried out using inorganic acids, organic acids, and bases as leaching media. Besides, auxiliary measures such as ultrasonic and mechanochemical are also used to enhance the leaching process.^15^^,^^16^^,^^17^^,^^18^^,^^19^^,^^20^ Hydrometallurgy recovery is more efficient and simpler to operate, but it will produce large quantities of waste acids and bases that will cause secondary pollution.^21^^,^^22^^,^^23^ Biometallurgy is probably the most environmentally friendly, but it has slow kinetics, high toxicity to micro-organisms, and a relatively low leaching efficiency at high leaching concentrations.^24^^,^^25^^,^^26^^,^^27^^,^^28^ Electrochemistry treatments become more and more widely used in batteries recovery, but most of them are done in aqueous solutions, which often generally require the aid of acids or bases for leaching.^29^^,^^30^^,^^31^^,^^32^ S.Y. Zhou et al. used circular a spent LiCoO_2_ electrode plate as the cathode, a platinum plate as the anode, and DL-malic acid as the electrolyte to recover spent LiCoO_2_. The leaching efficiencies of 97.25% for Li and 96.21% for Co could be obtained by electrolytic leaching.^32^ During the electrochemical recovery process in molten salts, electrons can be manipulated to drive the reduction, and thus the reduction of different substances can be controlled by adjusting the applied electrode potential to improve the efficiency of the reaction.^33^^,^^34^ At the same time, molten-salt systems have high ionic conductivities and wide electrochemical windows, making them excellent electrolytes for extraction and refining metals/alloys, energy storage, and materials synthesis.^35^ Zhang et al. used molten-salt electrolysis to reduce LiCoO_2_ to Li_2_O and Co/CoO in molten Na_2_CO_3_-K_2_CO_3_ at 750°C and the recovery efficiencies of Li and Co were 85% and 99%, respectively.^33^ At present, the mainstream method of dismantling method battery materials in the industry is crushing-flotation-sieving, thus separating the battery shell, copper foil, aluminum (Al) foil, plastic film, and black powder (mixture of the cathode and anode materials), among which further flotation is required if the black powder is to achieve the separation of cathode and anode materials.^36^^,^^37^^,^^38^ The existing recycling methods of LiCoO_2_ batteries using molten-salt electrochemistry do not consider the simultaneous recovery of the cathode and anode materials. In molten-salt electrochemical recovery of LiCoO_2_, the chemical bonds of LiCoO_2_ are broken electrochemically to separate Li and Co. Due to the insolubility of Co in the molten salt, the resulting solid product can be separated from the molten salt. In this way, the impurities in the anode material can be further removed, so the anode material can be recycled.

In this paper, a molten-salt electrochemical method was used to recover the cathode and anode of spent LiCoO_2_ batteries in NaCl-Na_2_CO_3_ molten salt. Correspondingly, the effects of various electrolysis parameters, such as voltages, time, intensity of pelletizing pressure, and other parameters on the products were investigated. The mechanism of recovery was analyzed and finally, the recovered graphite was reused. Using molten-salt electrolysis to co-recover the cathode and anode materials of spent LiCoO_2_ batteries can shorten the battery recycling process and save the recycling cost. The method also realizes the recycling of spent LiCoO_2_ batteries without using strong acids and bases.

## Results and discussion

### Thermodynamic analysis and electrochemical measurement

The standard equilibrium potential (Δ*E*) of an electrochemical reaction was obtained from the Gibbs free energy change (Δ*G*) of its reaction, as shown in Equation 1.

(Equation 1) $$\Delta E=-\frac{\Delta G}{nF}$$

where *n* is the number of molars of electrons transferred by the reaction and F is the Faraday constant (96485 C/mol).

**Figure 1:**
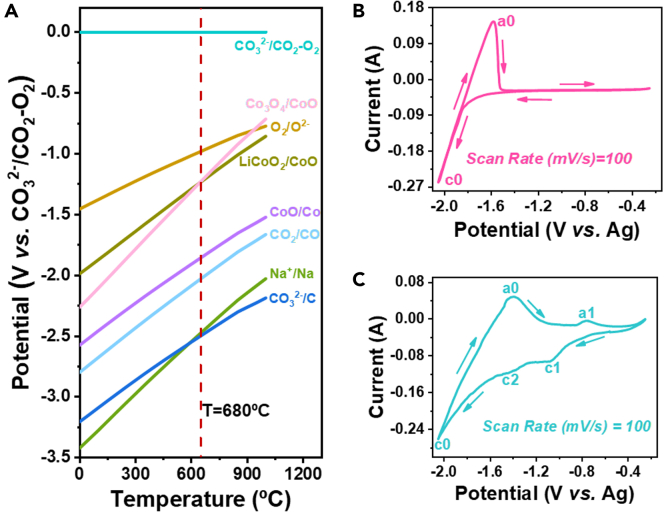
Thermodynamic data and cyclic voltammograms data with a scan rate of 100mV s^-1^ at 680℃ in molten NaCl-Na_2_CO_3_ Potential versus temperature curves in molten NaCl-Na_2_CO_3_ (all thermodynamic data were obtained from HSC Chemistry 6.0) (A), cyclic voltammograms of Mo-blank electrode (B), and Mo-LiCoO_2_ electrode (C) with a scan rate of 100 mV s^−1^ at 680°C in molten NaCl-Na_2_CO_3_.

Thermodynamically, the inorganic salts that constitute the molten-salt electrolyte must be more stable than LiCoO_2_. This phenomenon assures the LiCoO_2_ reduction if the decomposition voltage of LiCoO_2_ was lower than that of the molten salts (NaCl and Na_2_CO_3_). From Figure 1A, it could be seen that the reductive potentials of both LiCoO_2_ and cobalt oxides were more positive than that of NaCl and Na_2_CO_3_. Therefore, LiCoO_2_ can be converted to Co by electrolysis, whose process contains the preferentially production of cobalt oxide, and then the Co metal. Since the voltage difference of Co and Na generation is about 0.6 V, the generation of side reactions could be suppressed by controlling the voltages.

As shown in Figure 1B, a pair of redox peaks (c0/a0) correspond to the precipitation and dissolution of alkali metal ions on the Mo electrode in the molten NaCl-Na_2_CO_3_, respectively. When the mixture of LiCoO_2_ and graphite powders were loaded onto the Mo rod as the working electrode, two reductive peaks c1 and c2 appear at −0.9 V (vs. Ag/Ag^+^) and −1.3 V (vs. Ag/Ag^+^) corresponding to the reduction of LiCoO_2_ and cobalt oxide (Figure 1C), respectively. The peaks of c1 and c2 were the proofs of the stepwise reductive process of LiCoO_2_, i.e., LiCoO_2_ was reduced to cobalt oxide first, followed by the reduction of cobalt oxide to Co. Therefore, the type of electrolysis products could be controlled by changing the voltages, i.e., the target product Co could be obtained at a specific potential.

### Electrolysis

**Figure 2:**
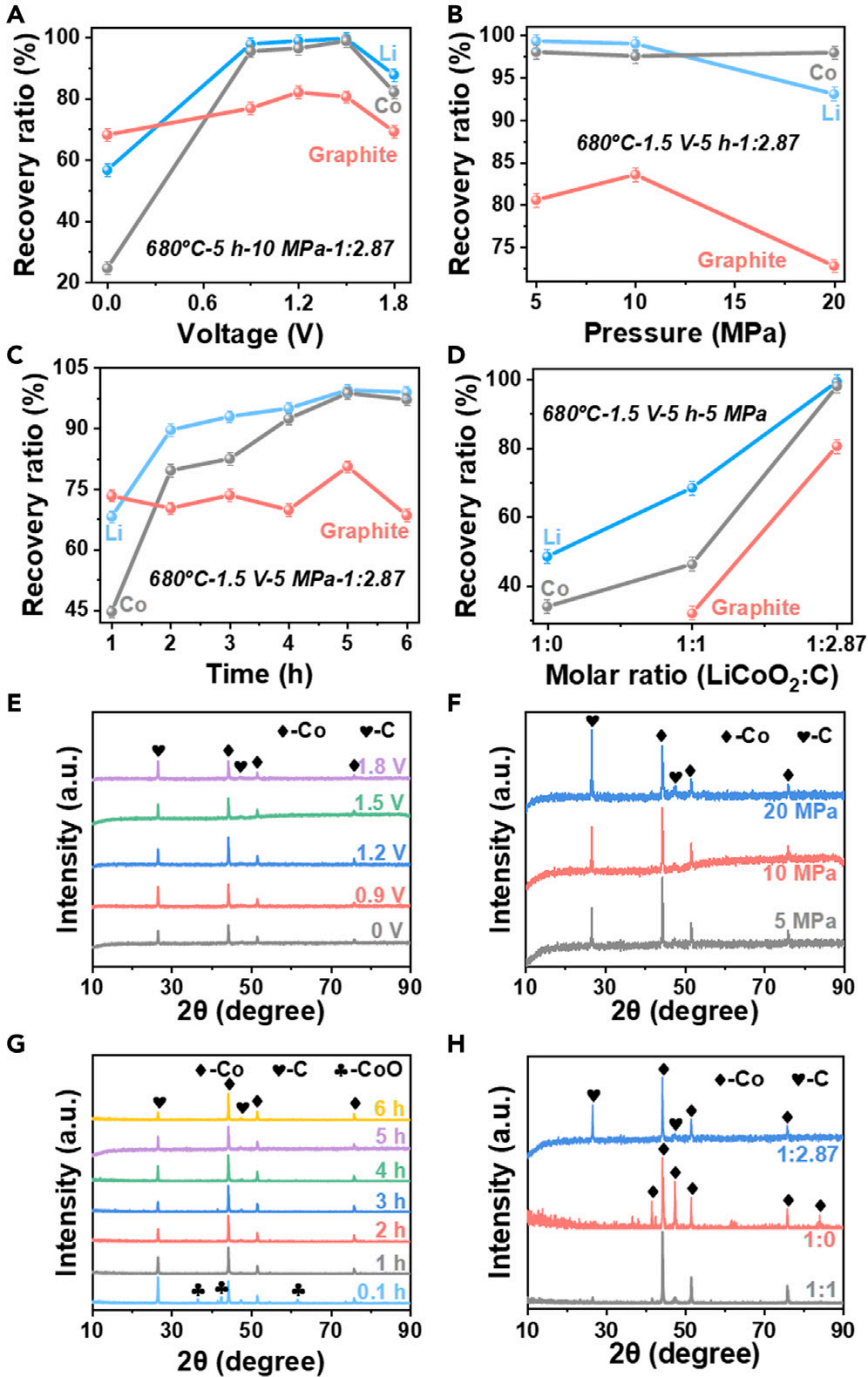
Recovery efficiencies and XRD patterns of magnetically separated fraction Recovery efficiencies of each substance for different electrolytic voltages (A), intensity of pelletizing pressure of pelletizing (B), electrolysis times (C), and pelletizing molar ratios (spent LiCoO_2_: spent graphite) (D), and XRD patterns of magnetically separated fraction for different electrolytic voltages (680°C, 5 h, 10 MPa, molar ratio of LiCoO_2_:C = 1:2.87) (E), intensity of pressures of pelletizing (680°C, 1.5 V, 5 h, molar ratio of LiCoO_2_:C = 1:2.87) (F), electrolytic times (680°C, 1.5 V, 5 MPa, molar ratio of LiCoO_2_:C = 1:2.87) (G), and pelleting molar ratios of spent LiCoO_2_ to spend graphite (680°C, 1.5V, 5 h, 5 MPa) (H) at 680°C in molten NaCl-Na_2_CO_3_.

From Figure 2A, when intensity of pelletizing pressure of the cathode material was 10 MPa and the electrolysis time was 5 h, the recovery efficiencies of Li and Co were the highest at 99.0% and 98.8% by constant-voltage electrolysis at 1.5 V. As the voltage increased, the electron transfer rate increased with a limited rate. When the electrolysis voltage was increased from 0.9 to 1.5 V, the recovery efficiency of Li did not increase significantly due to the limited reaction rate. In other words, the mass transfer rate in the molten salt could not keep up with the electron transfer rate provided by the voltage. However, the recovery efficiency of Li decreased significantly when the voltage reached 1.8 V. This may be attributed to the fact that the higher voltage could cause side reactions that disturb the target reaction. According to the product information, the impurities contained Mg and Al. At 680°C and an electrolysis voltage of 1.62 V, the electrochemical reaction is as follows: 2MgCl = 2Mg + Cl_2_ (g). At 680°C and an electrolysis voltage of 1.78 V, the electrochemical reaction is as follows: 2AlCl_3_ = 2Al + 3Cl_2_ (g).

The highest recovery efficiencies of Li and Co of 99.3% and 98.1% were achieved when intensity of pelletizing pressure on the cathode raw material was 5 MPa (seeing in Figure 2B). While intensity of pelletizing pressure at 20 MPa resulted in the lowest recovery efficiencies of Li and graphite at 93.1% and 72.9%. This distinction was ascribed to the penetration extent of molten salt into the cathode sheet, which largely affected the mass transfer efficiency. Generally, the electrolysis reaction on the cathode required sufficient contact between the cathode and the molten salt to ensure the transfer of electrons. The excessive intensity of pelletizing pressure not only affected the electrolysis efficiency but also delayed the Li^+^ entrance to the molten salt. The latter affected the mass transfer efficiency and further reduced the efficiency of the electrolysis reaction.

When the intensity of pelletizing pressure of the cathode material was 5 MPa at a voltage of 1.5 V, the recovery efficiencies of Li and Co gradually increased with the increase of electrolysis time until it stabilized (Figure 2C). When electrolysis occurred in the beginning, the reactants were very sufficient and the reactants on the surface layer of the cathode were more easily in contact with the molten salt. It was beneficial for mass transfer, so the recovery efficiency of Li increased rapidly. With the reaction proceeding, the reactants gradually decreased and the concentration of Li in the molten salt gradually increased, and it was more difficult for the cathode to contact the molten salt internally. This phenomenon caused the sluggish reaction rate corresponding to the flat of Li recovery efficiency. Therefore, 5 h was the optimal constant electrolysis time, in which the recovery efficiencies of Li, Co, and graphite were 99.0%, 98.8%, and 80.6%, respectively.

The molar ratio of the cathode and anode materials obtained after the disassembly of the spent LiCoO_2_ batteries is 1:2.87. From Figure 2D, it could be seen the lowest recovery efficiencies of Li and Co (48.5% and 34.0%) were obtained after electrolysis at a molar ratio of LiCoO_2_ to graphite of 1:0. At 680°C, the recovery efficiencies of Li obtained after electrolysis were 99.34% and 68.47% when the molar ratio of LiCoO_2_ to graphite were 1:2.87 and 1:1, respectively. The cathode mixture materials made of LiCoO_2_ and graphite reacted a partial carbothermal reduction reaction at 680°C, which was more favorable to the reduction of LiCoO_2_ under the condition of sufficient C. Compared with only LiCoO_2_ powder, the mixtures of graphite and LiCoO_2_ as the cathode could not only realize the simultaneous recovery of spent LiCoO_2_ cathode and anode materials but also improve the conductivity of the cathode in favor of faster electrolysis reaction efficiency. As the carbothermal reduction reaction of LiCoO_2_ generated CO_2_ gas, a fine pore structure was formed inside the cathode material, which was conducive to the full contact between the molten salt and LiCoO_2_ on the cathode. This could improve the electrolysis reaction efficiency. In this experiment, the recovery efficiencies of Li and Co reached 99.3% and 98.1%, respectively, and the recovery efficiency of graphite was 80.6% when the mixed spent electrode materials with a molar ratio of 1:2.87 was used as the electrolytic cathode. The recovery of spent cathode and anode electrodes together not only eliminated the step of separating the black powder again when the battery was disassembled in the industry but also realized the recovery of the spent cathode and anode electrodes.

The magnetically separated products obtained by electrolysis at 0.9,1.2, 1.5 V (5, 10, and 20 MPa), and 1.8 V for 1–6 h were Co, and no cobalt oxides were present (seeing in Figures 2E–2H). There was residual cobalt oxide in the products from magnetic separation at 1.5 V for 0.1 h with the intensity of pelletizing pressure 5 MPa (Figure 2G). Cobalt oxide exhibits para-magnetism and anti-magnetism at different temperatures, while Co has strong ferromagnetism. It is easily separated Co from graphite by magnetic separation. The product of electrolysis under suitable conditions was Co which facilitated the magnetic separation of the metal from the graphite.

**Figure 3:**
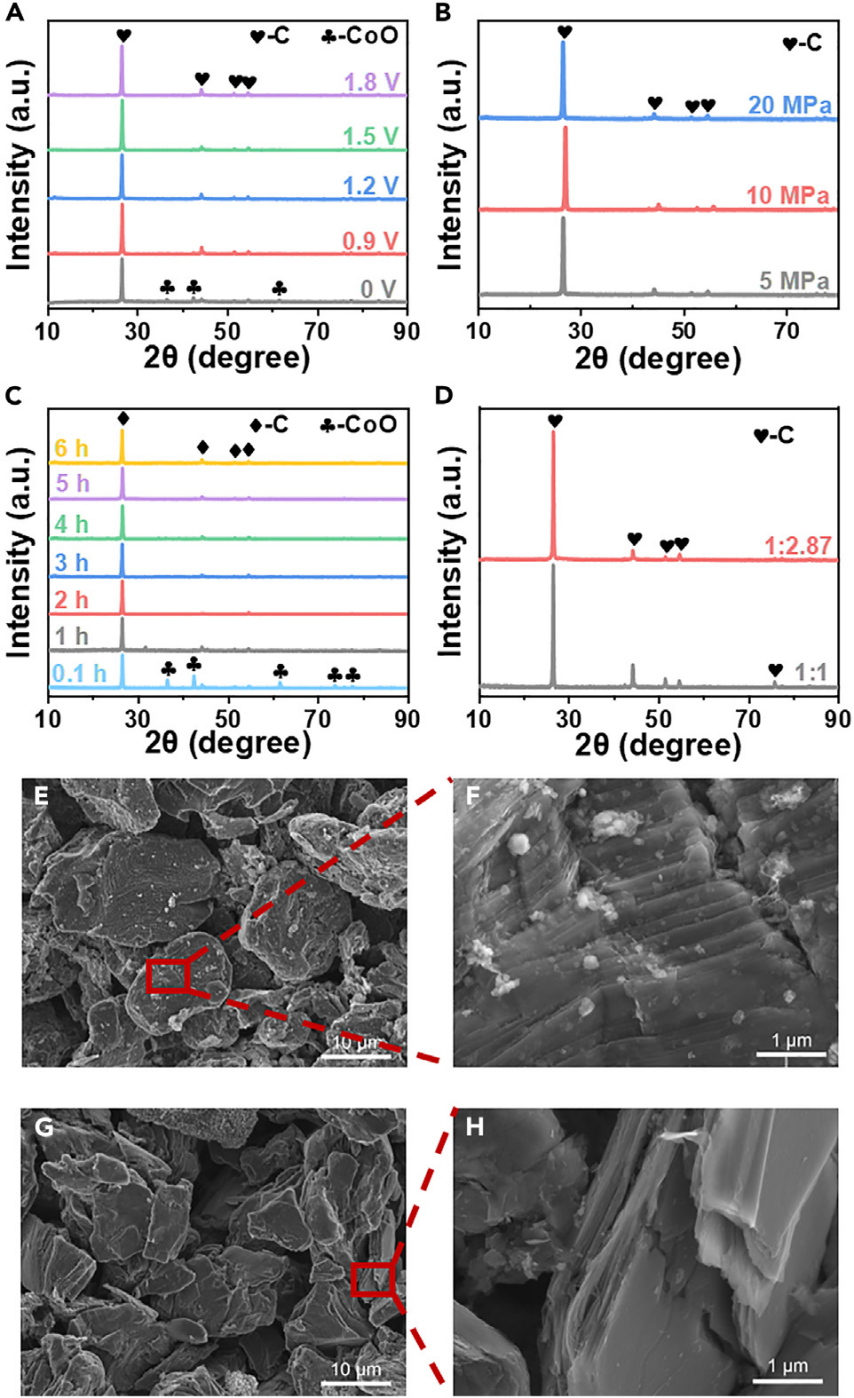
XRD patterns of magnetically separated residual material and SEM images of the spent and recovered graphite XRD patterns of magnetically separated residual material for different electrolytic voltages (680°C, 5 h, 10 MPa, molar ratio of LiCoO_2_:C = 1:2.87) (A), intensity of pressures of pelletizing (680°C, 1.5 V, 5 h, molar ratio of LiCoO_2_:C = 1:2.87) (B), electrolytic times (680°C, 1.5V, 5 MPa, molar ratio of LiCoO_2_:C = 1:2.87) (C), and pelleting molar ratios of spent LiCoO_2_to spend graphite (680°C, 1.5 V, 5 h, 5 MPa) (D) at 680°C in molten NaCl-Na_2_CO_3_, and SEM images of the spent graphite (E and F) and recovered graphite (G and H).

The remaining product of magnetic separation after electrolysis at 1.5 V-0.1 h is cobalt oxide and graphite (Figure 3A). The remaining electrolytic products after magnetic separation at 0.9, 1.2, 1.5 (5, 10, and 20 MPa), and 1.8V for 5 h and 1.5V for 1 ∼ 5h were pure graphite (Figures 3A-3D). In summary, under suitable electrolytic conditions, pure recycled graphite material could be obtained by magnetic separation. As could be seen from Figures 3E–3H, the sizes of the recycled graphite particles did not differ from those of the spent anode. There were some small particles on the surface of spent graphite particles, mainly due to the existence of several impurities, such as binder, electrolyte, and activated carbon.^39^^,^^40^^,^^41^^,^^42^ As the lithium embedding and de-lithiation process continues, the graphite layer spacing will continue to expand until it reaches a certain limit, so the capacity will gradually increase until it stabilizes. As the battery is charged and discharged over time, the graphite negative electrode expands to such an extent that its structure is permanently damaged and cannot be recovered, causing the lattice structure to collapse. The lattice collapse leads to structural rupture and the nanoparticles detached from the graphite negative electrode become attached to the surface of the micron-sized graphite particles together with the binder, electrolyte, and conductive agent. Impurities are no longer observed on the regenerated graphite surface, and the surface tends to be smooth with a perfect graphite morphology. These results can be attributed to the release of internal stresses in the spent graphite during the recycling process and the lattice tends to be fully restored. The recycled graphite became pure and maintained the laminar structure, which was suitable for the LIBs’ anode.

### Mechanism analysis

**Figure 4:**
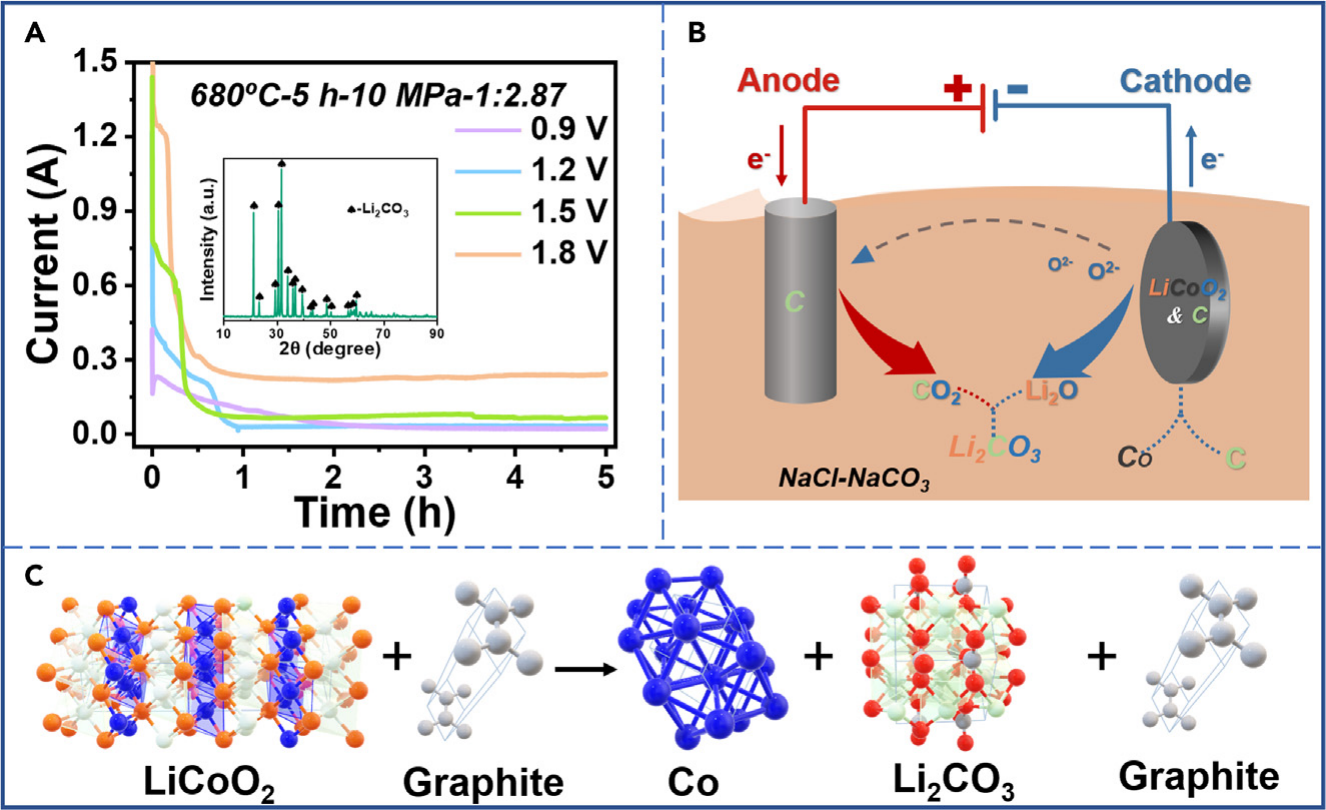
I-t curves, experimental reaction mechanism diagram, and structure chart of raw materials and products I-t curves and XRD image of the product after leaching and filtration of the molten-salt after electrolysis (molar ratio of LiCoO_2_:C = 1:2.87) (A), experimental reaction mechanism diagram (B), and structure chart of raw materials and products (C) at 680°C in molten NaCl-Na_2_CO_3_.

Observing Figure 4A, the current plateau was much smaller at 0.9 V compared to 1.8 V, which may be related to the slower reduction ratio at lower voltages. The product after electrolysis, leaching, and filtrating of the molten salt was Li_2_CO_3_. At constant voltage electrolysis, the first current plateau of each voltage was caused by the reduction of LiCoO_2_ to cobalt oxide. The processes remained for 39 min, 18.5 min, and 9 min at 1.2V, 1.5V, and 1.8V, respectively. Then, the reduction entered another rate-limiting step. The second current plateau of each voltage was generated by the reduction of cobalt oxide to Co. The subsequent slow decrease of current was due to the fact that fewer reactants are in contact with the molten salt, and the determinant of the reduction reaction rate gradually changed from the conduction of electrons to ion diffusion. The electrolysis curve shows a slow decrease in current until it finally stabilized at a certain current value. As shown in the reaction mechanism diagram in Figures 4B and 4C, during the electrolysis experiment, LiCoO_2_ on the cathode got electrons to be reduced to cobalt oxide or Co. Correspondingly, the resulting O^2−^ entered the molten salt and then generate CO_2_ by losing electrons on the graphite anode. Finally, residual Li_2_O entered the molten salt in the form of Li_2_CO_3_. The possible electrochemical reactions are as follows.

Cathode: 2LiCoO_2_ + 2e^−^ = 2CoO + Li_2_O + O^2−^ and CoO + 2e^−^ = Co + O^2-^
Anode: C + 2O^2−^ - 4e^−^ = CO_2_
Molten-salt: CO_2_ + Li_2_O = Li_2_CO_3_
Total reaction: 4LiCoO_2_ + 3C = 4Co + 2Li_2_CO_3_ + CO_2_ (g)
or
4 LiCoO_2_ + C = 4CoO + Li_2_CO_3_ + Li_2_O and 2CoO + C = 2Co + CO_2_ (g)

### Electrochemical performance of recovered graphite in LIBs

**Figure 5:**
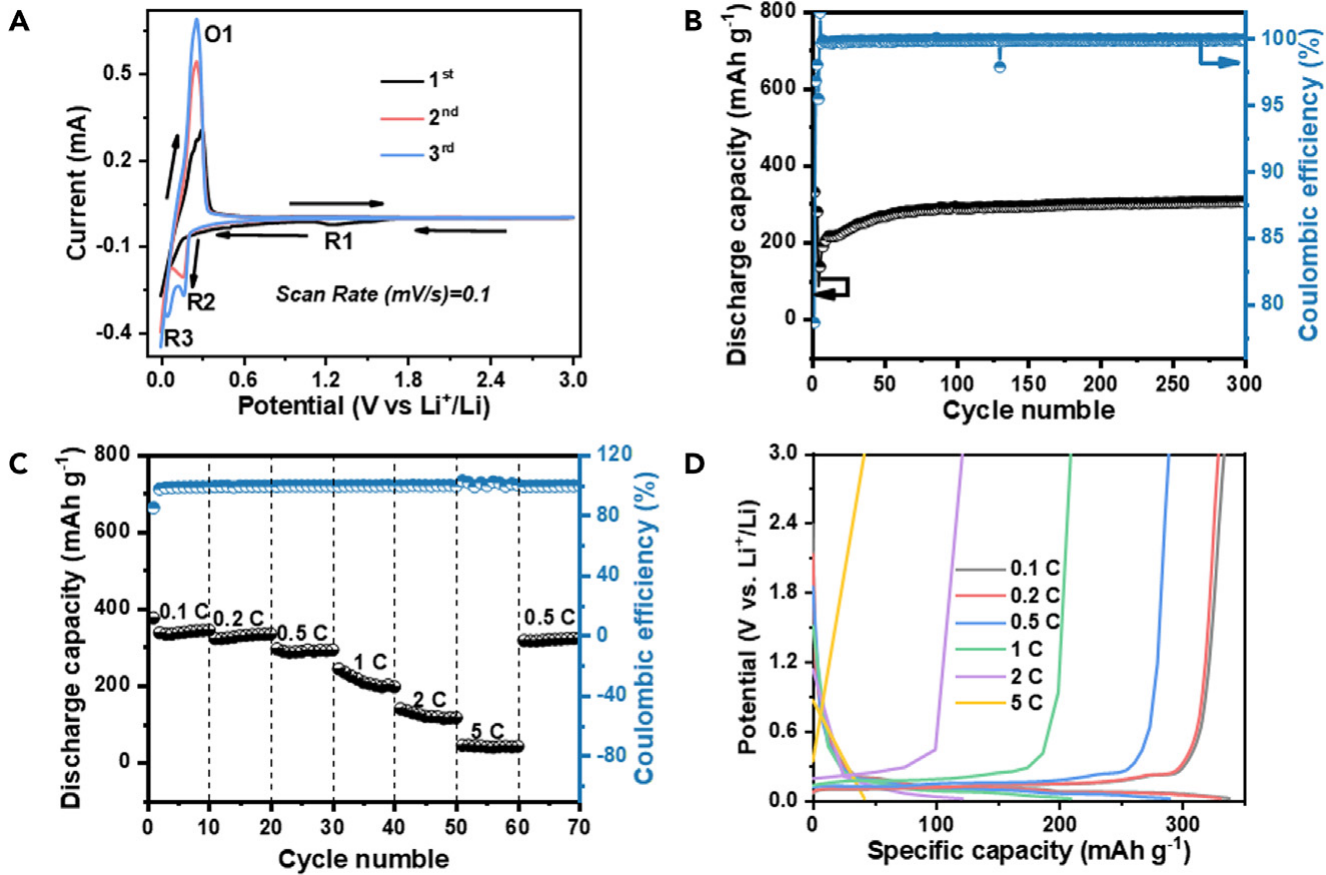
Cyclic voltammetry curves of graphite cell, cycling stability, rate performance, and potential-capacity plots at different rates Cyclic voltammetry curves of graphite cells at a sweep rate of 0.1 mV s^−1^ (A), cycling stability for 300 cycles at 1 C (B), rate performance (C), and potential-capacity plots at different rates (D) of the regenerated graphite button.

The recovered graphite could be reused as LIBs’ anode, and the electrochemical performances were evaluated. Figure 5A shows the CV curves of the recovered graphite in a graphite||Li half-cell. The peak R1 at around 1.3 V corresponded to the formation of the solid electrolyte interface (SEI) and disappeared in subsequent cycles, which means that SEI had become stable. The peaks of R2 and R3 were related to the lithiation process of recovered graphite. The reduction peak R3 appeared near 0.01 V in the first cycle and moved toward 0.2 V (peak R2) in the second and third cycles, indicating the embedding of Li^+^ in the graphite layer in the second and third cycles. The oxidation peak O1 was observed near 0.38 V in the first cycle, corresponding to the de-embedding of Li^+^ in the graphite layer, and moved to 0.3 V in the next two cycles, which can be attributed to the delayed penetration of the electrolyte into the electrode sheet. The peak current gradually increased from the first to the third cycle, indicating that the electrode was gradually activated and the reaction rate accelerated with charge/discharge cycles.

Figure 5B shows the cycling performance of the recovered graphite. The charge-discharge curve of recycled graphite at 1 C (assuming 1 C = 372 mA h^−1^) showed its first discharge-charge specific capacity of 266.1 mA h g^−1^. The first cycle resulted in an irreversible capacity of 58.0 mA h g^−1^ due to the formation of the SEI, so the second cycle showed a rapid decrease in capacity compared to the first cycle. For the first three cycles, the battery was activated by charging and discharging at 0.1C. And from the fourth cycle onwards, the battery was tested at 1C, so it appears that the battery capacity attenuated rapidly in the first few cycles. As the lithium embedding and de-lithiation process continues, the graphite layer spacing will continue to expand until it reaches a certain limit, so then the capacity will gradually increase until it stabilizes. As shown in Figure 5C, the recovered graphite exhibited excellent rate performance. The discharge-charge capacities at 0.1, 0.2, 0.5, 1, and 2C were 321.2, 321, 294.7, 243.3, and 140.4 mA h g^−1^, respectively. The discharge capacity remained at about 316.2 mA h g^−1^ at 0.5 C after the rate capability test, indicating that the revered graphite had good stability after high-rate cycling. Figure 5D shows the charge/discharge curves (0.5 ∼ 5C) of recovered graphite, which shows that recovered graphite exhibited good reversibility in the graphite||Li half-cell. From the above results, the recovered graphite showed excellent battery performance for LIBs.

### Conclusions

**Figure 6:**
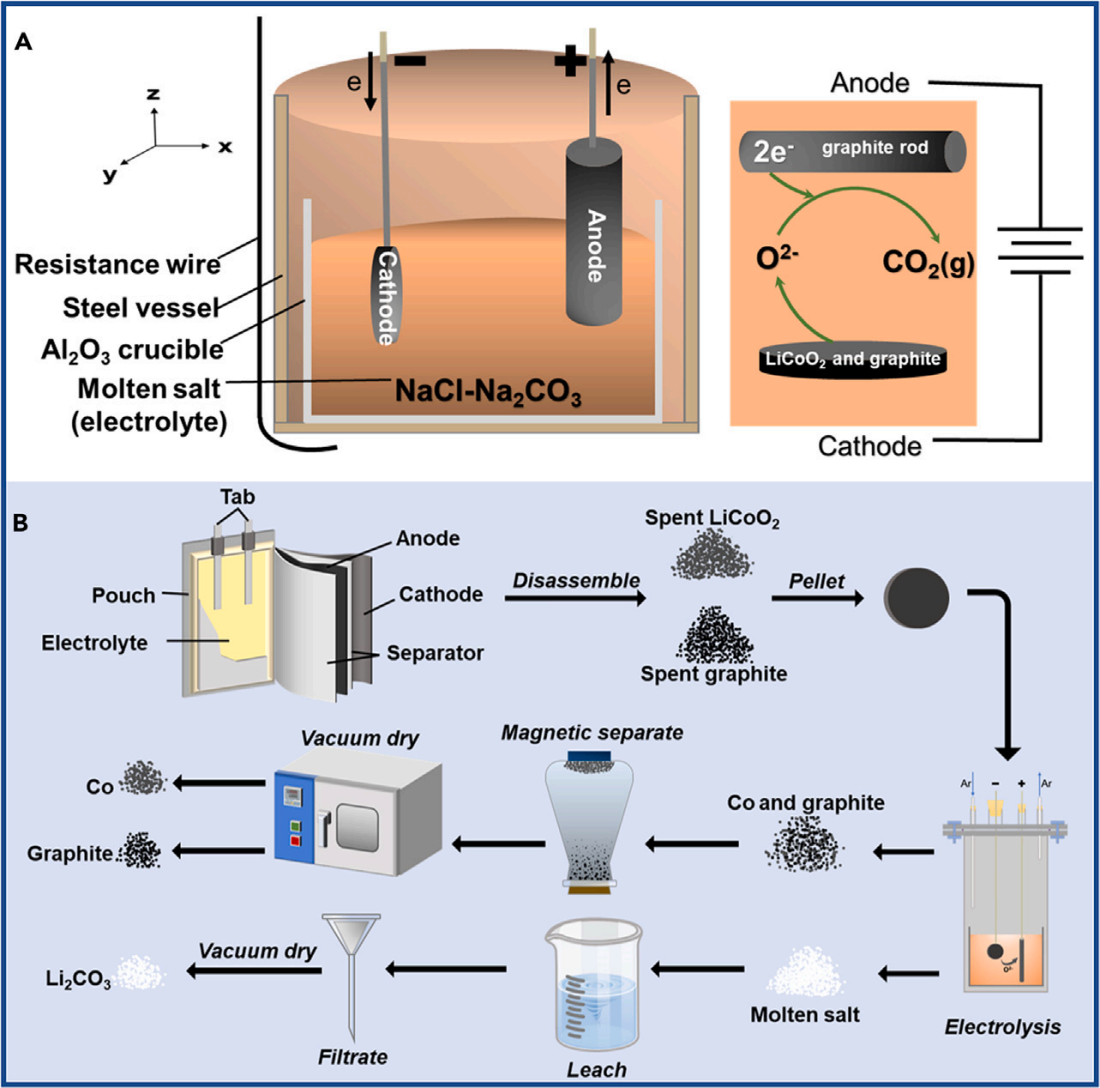
Experimental apparatus and flow chart Experimental apparatus (A) and flow chart (B) for the recovery of Co, Li, and graphite from spent LiCoO_2_ batteries.

The experimental apparatus and flow chart for this work were shown in Figure 6. Molten-salt electrolysis has been demonstrated as an efficient way to co-recover the cathode and anode of spent LiCoO_2_ batteries in molten NaCl-Na_2_CO_3_. At the optimal electrolysis conditions (1.5 V for 5 h and at 680°C), the recovery efficiencies of Li, Co, and graphite reached 99.3%, 98.1%, and 83.6%, respectively. The use of electrons instead of chemical reagents to destroy the crystal structure of LiCoO_2_ achieved the separation of Li, Co, and graphite. This electrochemical process greatly reduced the secondary waste. In addition, the relatively low operational temperature avoided the direct carbothermic reduction and co-recycled the Co and graphite. The recovered graphite showed similar electrochemical performance as the original commercial graphite. Further, the molten-salt electrochemical method can be used for the recovery of other types of spent LIBs with the aim to reduce secondary waste by using renewable electricity as the clean agent and driving force.

### Limitations of the study

**Figure 7:**
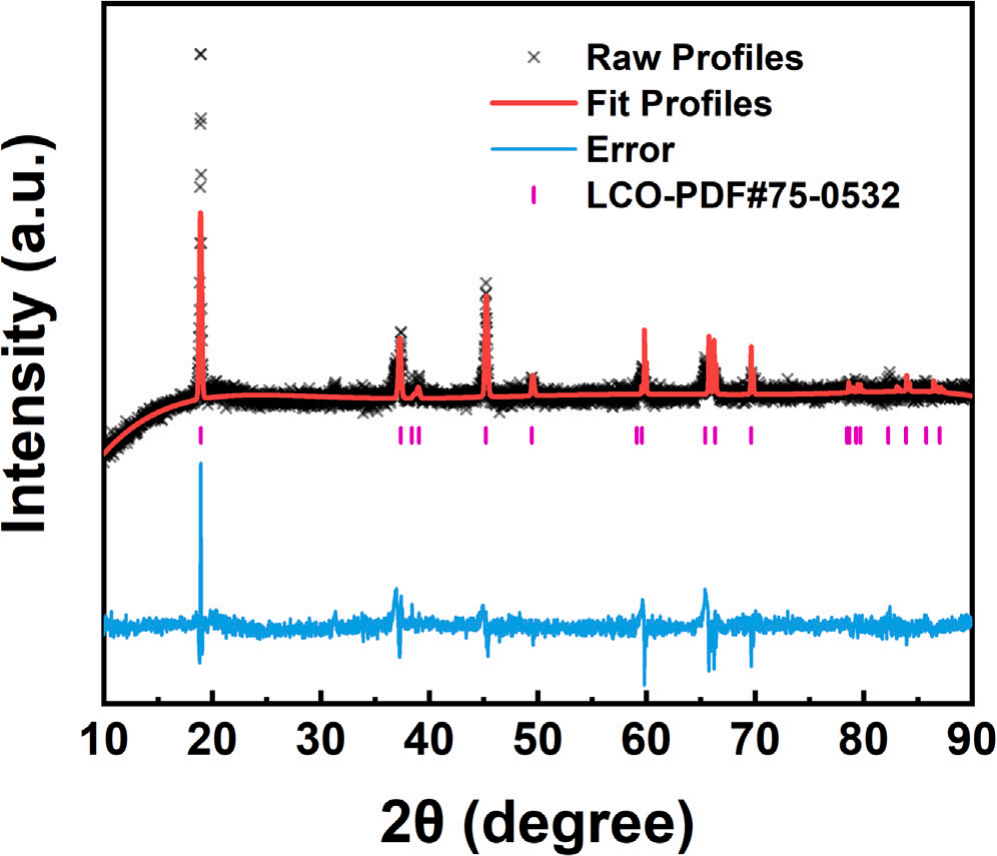
XRD Rietveld refinement of the cathode material of spent LiCoO_2_ batteries

There are some limitations to this study. We collected the cathode material of spent LiCoO_2_ batteries and performed X-ray diffraction spectroscopy (XRD) inspection and XRD Riedveld refinement. As shown in Figure 7 and Table 1, the wR of the refined result was 2.232%, indicating that the spent cathode powder primarily consists of LiCoO_2_.^43^ The element content of cathode of the spent LiCoO_2_ batteries was tested by inductively coupled plasma optical emission spectrometer (ICP-OES) and X-ray fluorescence spectrometer (XRF). The test results are shown in Table 2. The spent cathode powder consists predominantly of LiCoO_2_, representing 98.8813wt %. The primary impurity within the spent cathode powder is Al, which originates from a small quantity of aluminum foil scraped along with the spent cathode powder. In this study we have only discussed the LiCoO_2_ recovery process and have not discussed the effect of other impurities on the reaction process.

**Table 1:** Cell parameters of sample for the cathode material of spent LiCoO_2_ batteries

Sample	V/Å^3^	a/Å	b/Å	c/Å	α/°	β/°	γ/°	wR/%
LiCoO_2_	96.337	2.814	2.814	14.048	90	90	120	–
Spent LiCoO_2_	95.201	2.81126	2.81126	13.90946	90	90	120	2.232

**Table 2:** Composition of spent cathode powder

Element	Co	O	Li	Al	Ti	P	Fe	Mg	S	C	Si
Wt %	59.5406	32.3286	7.0121	0.5735	0.1298	0.1126	0.0861	0.0818	0.0552	0.0416	0.0376

There is a limitation that the experiment was not amplified. As only 0.26 g of graphite was used in each experiment, a very small amount of weight can cause a large change in recovery. In our experiments, we separated the graphite and placed it in a beaker to dry, then scraped it out of the beaker and weighed it again. If the inner wall of the beaker is no longer smooth, some graphite will be lost, introducing an error due to handling problems, and if the beaker is very smooth, this error is greatly reduced. As speculated, the total graphite recovery in the manuscript is slightly lower than the actual recovery. So, there is some error in the graphite recovery ratio in Figure 2, but the data still have some general reference value.

## STAR★Methods

### Key resources table

**Table:** 

REAGENT or RESOURCE	SOURCE	IDENTIFIER
**Chemicals, peptides, and recombinant proteins**		

Na_2_CO_3_	Tianjin Damao Chemical Reagent Factory, China	CAS497-19-8
NaCl	Tianjin Damao Chemical Reagent Factory, China	CAS7647-14-5
HNO_3_	Sinopharm Chemical Reagent Co., Ltd, China	CAS7697-37-2
Argon (Ar) gas	Shuntai, Co., Ltd, China	CAS7440-37-1
acetylene black	Yingze District, Taiyuan City, the source of the battery sales department, China	N/A
polyvinylidene difluoride	Shanghai Macklin Biochemical Co., Ltd	CAS9002-84-0

**Software and algorithms**		

GSAS-2	https://subversion.xray.aps.anl.gov/trac/pyGSAS	N/A

### Resource availability

#### Lead contact

Further information and requests for resources and reagents should be directed to and will be fulfilled by the lead contact, Huayi Yin (yinhuayi@whu.edu.cn).

#### Materials availability

This study did not generate new unique reagents.

### Experimental model and study participant details

There are no experimental model and study participant details to be reported.

### Method details

#### Materials and pretreatment

Sodium carbonate (Na_2_CO_3_, AR, 99%) and sodium chloride (NaCl, AR, 99.8%) were purchased from Tianjin Damao Chemical Reagent Factory. The spent LiCoO_2_ batteries were obtained from the local electronic market in Shenyang, Liaoning Province, China. HNO_3_ (65% ∼ 68%) was purchased from Sinopharm Chemical Reagent Co., Ltd. Argon (Ar) gas, 99.99%, was purchased from Shuntai, Co., Ltd.

The spent LiCoO_2_ batteries were placed in saturated NaCl solution for 24 h to be thoroughly discharged and then dried under vacuum at 60°C for 12 h. The dried batteries were disassembled in a fuming hood, and the obtained cathode electrodes were pyrolyzed at 450°C for 1.5 h to remove the electrolyte and organic binder. Then the obtained cathode was roasted in the air at 800°C for 2 h to remove the acetylene black.^44^^,^^45^ The degraded LiCoO_2_ powder was obtained by scraping off the Al foil and removing the residual Al foil fragments using an 800-mesh sieve. We collected the obtained the cathode material of spent LiCoO_2_ batteries and performed X-ray diffraction spectroscopy (XRD) inspection and XRD Rietveld refinement. As shown in Figure 7 and Table 1, the wR of the refined result was 2.232%, indicating that the spent cathode powder primarily consists of LiCoO_2_.^43^

The disassembled anode electrodes were placed in deionized water and soaked for 12 h. After the anode electrode material was separated from the copper foil, the collected anode material was washed and filtered several times using deionized water, and then placed in a vacuum environment and dried under vacuum at 60°C for 12 h.

#### Cyclic voltammetry of LiCoO_2_ powder in molten NaCl-Na_2_CO_3_ salt

Firstly, a mixture of NaCl-Na_2_CO_3_ powder (NaCl/Na_2_CO_3_ molar ratio of 0.577:0.423) was dried under vacuum at 200°C for 12 h to remove moisture. The Al_2_O_3_ crucible (Φ=100 mm, H=100 mm) was filled with anhydrous NaCl-Na_2_CO_3_ mixtures as the electrolyte, and the Al_2_O_3_ crucible was sealed in a stainless-steel reactor which was heated by a vertical resistance furnace. Then, the temperature was increased to 680°C at a heating rate of 5°C min^-1^ to melt the salts with the protection of continuous Ar flow. Finally, the cyclic voltammetry (CV) test was first performed using a three-electrode system controlled by an electrochemical workstation (CHI 1140C, CH Instrument Company, China) with a scan rate of 100 mV/s. An Ag wire was used as the reference electrode, and a graphite rod was equipped as the counter electrode. A Mo rod and Mo rod decorated with the recovered electrode materials (both cathode and anode) were applied as the working electrodes, respectively.

#### Constant voltage electrolysis of mixed LiCoO_2_ and graphite

In the same molten-salt system described above, the electrolysis was conducted by a two-electrode system. The system consisted of mixed spent electrode materials (used as cathode, surface area in contact with the molten salt is 3.69 cm^2^) and a graphite rod (used as anode, surface area in contact with the molten salt is 10.55 cm^2^) under the control of a high-precision battery performance test system (Neware CT-9004-5V5A-G4, Neware technology limited, China). The electrolysis was carried out at different voltages (0.9, 1.2, 1.5, and 1.8 V), different granulation intensities of strength (5, 10, and 20 MPa), different electrolysis times (0.1, 1, 2, 3, 4, 5, and 6 h), and different production molar ratios of LiCoO_2_ to graphite (1:1, 1:0, and 1:2.87) under constant voltage control. A constant temperature of 680°C was maintained throughout the electrolysis process. The experimental apparatus and flow chart for this work were shown in Figure 6.

#### Recovery of Co, Li, and graphite

The electrolysis products Co and graphite from the cathode were separated by magnetic separation and weighed individually for further characterization. The recovery efficiencies of graphite and Co ($R_{C}$ and $R_{\text{Co}}$) could be calculated by Equations 2 and 3, respectively.

(Equation 2) $$R_{C}=\frac{\text{the}\;\text{mass}\;\text{of}\;\text{the}\;\text{recovered}\;\text{graphite}}{\text{the}\;\text{mass}\;\text{of}\;\mathrm{graphite}\;\text{in}\;\text{the}\;\text{powder}\;\text{mixture}\;\text{of}\;{\text{LiCoO}}_{2}\;\text{and}\;\text{graphite}}$$

(Equation 3) $$R_{\text{Co}}=\frac{\text{the}\;\text{mass}\;\text{of}\;\text{the}\;\text{recovered}\;\text{Co}}{{\text{the}\;\text{mass}\;\text{of}\;\text{Co}\;\text{in}\;\text{the}\;\text{powder}\;\text{mixture}\;\text{of}\;\text{LiCoO}}_{2}\;\text{and}\;\text{graphite}}$$

After the electrolysis experiments, the molten salt was allowed to cool down, then put into an agate mortar, and used pestle to grind it well into the powder state. The obtained powder was put into deionized water at 90°C to dissolve NaCl and Na_2_CO_3_, and the undissolved Li_2_CO_3_ was obtained by washing and filtering several times. Finally, the obtained Li_2_CO_3_ powder was dried under vacuum at 60°C for 12 h for further characterization. To measure the concentration of Li^+^ in the molten salt after electrolysis, ∼0.5 g sample of molten salt was taken after each set of electrolysis experiments and dissolved in a 0.05 mol L^-1^ HNO_3_ solution at a solid-liquid ratio of 5 g L^-1^. After the salt was completely dissolved, the concentration of Li^+^ in the solution was measured by atomic absorption spectrometry (AAS). The recovery efficiency of Li^+^ ($R_{{\text{Li}}^{+}}$) can be calculated by Equation 4.

(Equation 4) $$R_{{\text{Li}}^{+}}=\frac{\text{the}\;\text{mass}\;\text{of}\;\text{Li}\;\text{in}\;\text{the}\;\text{recovered}\;{\text{Li}}_{2}{\text{CO}}_{3}}{\text{the}\;\text{mass}\;\text{of}\;\text{Li}\;\text{in}\;\text{the}\;\text{powder}\;\text{of}\;{\text{LiCoO}}_{2}}$$

#### Electrochemical performance of recovered graphite in LIBs

Regenerated graphite, acetylene black, and polyvinylidene difluoride (PVDF) were mixed and ground with a mass ratio of 8.5:0.5:1. N-methyl pyrrolidone (NMP) was added to the well-mixed material and stirred for 2 h to make a yogurt-like slurry. The prepared slurry was pasted to the copper foil and dried under vacuum at 80°C for 12 h. Then, the copper foil containing the slurry was cut into small discs with a diameter of 12 mm using a precision disc cutter (Shenzhen Kejing MSK-T10). Finally, the obtained electrode discs were transferred into an Ar-filled glove box (M. Braun) with water and oxygen values below 0.1 ppm. The CR2032-type button half-cells were assembled in the glove box with the recycled graphite as the working electrode. Li foil was used as the counter electrode, and Celgard 2300 porous membrane was chosen as the separator. The assembled cells were allowed to stand for 24 h at 24°C to ensure that the electrolyte could fully wet the electrodes. Constant current charge/discharge tests of the half-cells were performed at 24°C in a battery test system (Wuhan LANHE CT2001A) with a voltage range of 0.01 ∼ 3 V (vs. Li^+^/Li). Cyclic voltammetry (CV) tests were performed at 24°C using the CHI 1140C electrochemical workstation with a voltage range of 0.01 ∼ 3 V (vs. Li^+^/Li) and a scan rate of 0.1 mV/s.

#### Material characterization

The concentration of Li^+^ was measured by atomic absorption spectroscopy (AAS, TAS-990). All materials were characterized by scanning electron microscopy (SEM, FEI Sirion field emission) and X-ray diffraction spectroscopy (XRD, Shimadzu X-ray 6000, Cu Kα radiation, λ = 1.5405 Å).

### Additional resources

There are no additional resources to be reported.

## Data Availability

• The data reported in this paper will be shared by the lead contact upon request. • This paper does not report original code. • Any additional information required to reanalyze the data reported in this paper is available from the lead contact upon request.

## References

[1] Tarascon J.-M., Armand M. (2001). Issues and Challenges Facing Rechargeable Lithium Batteries. Nature.

[2] Armand M., Tarascon J.-M. (2008). Building Better Batteries. Nature.

[3] Meshram P., Mishra A., Abhilash, Sahu R. (2020). Environmental Impact of Spent Lithium Ion Batteries and Green Recycling Perspectives by Organic Acids – A Review. Chemosphere.

[4] Lu B., Liu J., Yang J. (2017). Substance Flow Analysis of Lithium for Sustainable Management in Mainland China: 2007–2014. Resour. Conserv. Recycl..

[5] Seong W.M., Park K.-Y., Lee M.H., Moon S., Oh K., Park H., Lee S., Kang K. (2018). Abnormal Self-Discharge in Lithium-Ion Batteries. Energy Environ. Sci..

[6] Mossali E., Picone N., Gentilini L., Rodrìguez O., Pérez J.M., Colledani M. (2020). Lithium-Ion Batteries towards Circular Economy: A Literature Review of Opportunities and Issues of Recycling Treatments. J. Environ. Manage..

[7] Liu B., Jia Y., Li J., Yin S., Yuan C., Hu Z., Wang L., Li Y., Xu J. (2018). Safety Issues Caused by Internal Short Circuits in Lithium-Ion Batteries. J. Mater. Chem. A Mater..

[8] Zhang X., Li L., Fan E., Xue Q., Bian Y., Wu F., Chen R. (2018). Toward Sustainable and Systematic Recycling of Spent Rechargeable Batteries. Chem. Soc. Rev..

[9] Zhang B., Qu X., Chen X., Liu D., Zhao Z., Xie H., Wang D., Yin H. (2022). A Sodium Salt-Assisted Roasting Approach Followed by Leaching for Recovering Spent LiFePO_4_ Batteries. J. Hazard Mater..

[10] Li J., Lai Y., Zhu X., Liao Q., Xia A., Huang Y., Zhu X. (2020). Pyrolysis Kinetics and Reaction Mechanism of the Electrode Materials during the Spent LiCoO_2_ Batteries Recovery Process. J. Hazard Mater..

[11] Makuza B., Tian Q., Guo X., Chattopadhyay K., Yu D. (2021). Pyrometallurgical Options for Recycling Spent Lithium-Ion Batteries: A Comprehensive Review. J. Power Sources.

[12] Li M., Zhang B., Qu X., Cai M., Liu D., Zhou F., Xie H., Gao S., Yin H. (2022). A SiCl_4_-Assisted Roasting Approach for Recovering Spent LiCoO_2_ Cathode. ACS Sustain. Chem. Eng..

[13] Takacova Z., Orac D., Klimko J., Miskufova A. (2023). Current Trends in Spent Portable Lithium Battery Recycling. Materials.

[14] Li J., Wang G., Xu Z. (2016). Environmentally-Friendly Oxygen-Free Roasting/Wet Magnetic Separation Technology for in Situ Recycling Cobalt, Lithium Carbonate and Graphite from Spent LiCoO_2_/Graphite Lithium Batteries. J. Hazard Mater..

[15] Myoung J., Jung Y., Lee J., Tak Y. (2002). Cobalt Oxide Preparation from Waste LiCoO_2_ by Electrochemical–Hydrothermal Method. J. Power Sources.

[16] Yao Y., Zhu M., Zhao Z., Tong B., Fan Y., Hua Z. (2018). Hydrometallurgical Processes for Recycling Spent Lithium-Ion Batteries: A Critical Review. ACS Sustain. Chem. Eng..

[17] Li L., Chen R., Zhang X., Wu F., Ge J., Xie M. (2012). Preparation and electrochemical properties of re-synthesized LiCoO_2_ from spent lithium-ion batteries. Chin. Sci. Bull..

[18] Chen L., Tang X., Zhang Y., Li L., Zeng Z., Zhang Y. (2011). Process for the Recovery of Cobalt Oxalate from Spent Lithium-Ion Batteries. Hydrometallurgy.

[19] Ku H., Jung Y., Jo M., Park S., Kim S., Yang D., Rhee K., An E.-M., Sohn J., Kwon K. (2016). Recycling of Spent Lithium-Ion Battery Cathode Materials by Ammoniacal Leaching. J. Hazard Mater..

[20] Yang Y., Meng X., Cao H., Lin X., Liu C., Sun Y., Zhang Y., Sun Z. (2018). Selective Recovery of Lithium from Spent Lithium Iron Phosphate Batteries: A Sustainable Process. Green Chem..

[21] Nan J., Han D., Zuo X. (2005). Recovery of Metal Values from Spent Lithium-Ion Batteries with Chemical Deposition and Solvent Extraction. J. Power Sources.

[22] Zhao J., Zhang B., Xie H., Qu J., Qu X., Xing P., Yin H. (2020). Hydrometallurgical Recovery of Spent Cobalt-Based Lithium-Ion Battery Cathodes Using Ethanol as the Reducing Agent. Environ. Res..

[23] Li L., Lu J., Ren Y., Zhang X., Chen R., Wu F., Amine K. (2012). Ascorbic-Acid-Assisted Recovery of Cobalt and Lithium from Spent Li-Ion Batteries. J. Power Sources.

[24] Huang L., Liu Y., Yu L., Quan X., Chen G. (2015). A New Clean Approach for Production of Cobalt Dihydroxide from Aqueous Co(II) Using Oxygen-Reducing Biocathode Microbial Fuel Cells. J. Clean. Prod..

[25] Roy J.J., Cao B., Madhavi S. (2021). A Review on the Recycling of Spent Lithium-Ion Batteries (LIBs) by the Bioleaching Approach. Chemosphere.

[26] Sethurajan M., Gaydardzhiev S. (2021). Bioprocessing of Spent Lithium Ion Batteries for Critical Metals Recovery – A Review. Resour. Conserv. Recycl..

[27] Dolker T., Pant D. (2019). Chemical-Biological Hybrid Systems for the Metal Recovery from Waste Lithium Ion Battery. J. Environ. Manage..

[28] Calvert G., Kaksonen A., Cheng K., Van Yken J., Chang B., Boxall N. (2019). Recovery of Metals from Waste Lithium Ion Battery Leachates Using Biogenic Hydrogen Sulfide. Minerals.

[29] Li Z., He L., Zhu Y., Yang C. (2020). A Green and Cost-Effective Method for Production of LiOH from Spent LiFePO_4_. ACS Sustain. Chem. Eng..

[30] Garcia E.M., Santos J.S., Pereira E.C., Freitas M.B.J.G. (2008). Electrodeposition of Cobalt from Spent Li-Ion Battery Cathodes by the Electrochemistry Quartz Crystal Microbalance Technique. J. Power Sources.

[31] Li L., Chen R., Sun F., Wu F., Liu J. (2011). Preparation of LiCoO_2_ Films from Spent Lithium-Ion Batteries by a Combined Recycling Process. Hydrometallurgy.

[32] Zhou S., Zhang Y., Meng Q., Dong P., Yang X., Liu P., Li Q., Fei Z. (2021). Recycling of Spent LiCoO_2_ Materials by Electrolytic Leaching of Cathode Electrode Plate. J. Environ. Chem. Eng..

[33] Zhang B., Xie H., Lu B., Chen X., Xing P., Qu J., Song Q., Yin H. (2019). A Green Electrochemical Process to Recover Co and Li from Spent LiCoO2-Based Batteries in Molten Salts. ACS Sustain. Chem. Eng..

[34] Li H., Li H., Li C., Liang J., Yan H., Xu Z. (2021). Study on the Behavior of Electrochemical Extraction of Cobalt from Spent Lithium Cobalt Oxide Cathode Materials. Materials.

[35] Li M., Liu C., Ding A., Xiao C. (2023). A Review on the Extraction and Recovery of Critical Metals Using Molten Salt Electrolysis. J. Environ. Chem. Eng..

[36] Zhang T., He Y., Wang F., Ge L., Zhu X., Li H. (2014). Chemical and Process Mineralogical Characterizations of Spent Lithium-Ion Batteries: An Approach by Multi-Analytical Techniques. Waste Manag..

[37] Pagnanelli F., Moscardini E., Altimari P., Abo Atia T., Toro L. (2017). Leaching of Electrodic Powders from Lithium Ion Batteries: Optimization of Operating Conditions and Effect of Physical Pretreatment for Waste Fraction Retrieval. Waste Manag..

[38] Wang X., Gaustad G., Babbitt C.W. (2016). Targeting High Value Metals in Lithium-Ion Battery Recycling via Shredding and Size-Based Separation. Waste Manag..

[39] Liu D., Qu X., Zhang B., Zhao J., Xie H., Yin H. (2022). Alkaline Roasting Approach to Reclaiming Lithium and Graphite from Spent Lithium-Ion Batteries. ACS Sustain. Chem. Eng..

[40] Yu H., Dai H., Zhu Y., Hu H., Zhao R., Wu B., Chen D. (2021). Mechanistic Insights into the Lattice Reconfiguration of the Anode Graphite Recycled from Spent High-Power Lithium-Ion Batteries. J. Power Sources.

[41] Xiao J., Li J., Xu Z. (2017). Recycling Metals from Lithium Ion Battery by Mechanical Separation and Vacuum Metallurgy. J. Hazard Mater..

[42] Cheng Q., Marchetti B., Chen X., Xu S., Zhou X.-D. (2022). Separation, Purification, Regeneration and Utilization of Graphite Recovered from Spent Lithium-Ion Batteries - A Review. J. Environ. Chem. Eng..

[43] Toby B.H., Von Dreele R.B. (2013). GSAS-II: The Genesis of a Modern Open-Source All Purpose Crystallography Software Package. J. Appl. Crystallogr..

[44] Nie H., Xu L., Song D., Song J., Shi X., Wang X., Zhang L., Yuan Z. (2015). LiCoO_2_: Recycling from Spent Batteries and Regeneration with Solid State Synthesis. Green Chem..

[45] Zhang X., Xue Q., Li L., Fan E., Wu F., Chen R. (2016). Sustainable Recycling and Regeneration of Cathode Scraps from Industrial Production of Lithium-Ion Batteries. ACS Sustain. Chem. Eng..

